# Prosthetic Rehabilitation With Customized Nasal Prosthesis Following Partial Rhinectomy: A Case Report

**DOI:** 10.7759/cureus.69112

**Published:** 2024-09-10

**Authors:** Pinky Vincent, Sandhya M Raghavan, Rajesh C, Sreejith Sreekumar

**Affiliations:** 1 Prosthodontics, Government Dental College, Kottayam, IND; 2 Dentistry, PMS College of Dental Science and Research, Thiruvananthapuram, IND

**Keywords:** eyewear retention, nasal carcinoma, nasal prosthesis, polymethyl methacrylate, rhinectomy

## Abstract

Nasal prostheses have long served as critical solutions for patients who have lost part or all of their nose due to cancer, accidents, or congenital deformities. Discussed here is a case report of a patient with basal cell carcinoma who underwent partial rhinectomy and was rehabilitated with a nasal prosthesis fabricated with polymethyl methacrylate and retained with eyewear. The integration of nasal prostheses with eyewear frames not only enhances stability and comfort but also streamlines the daily routine of prosthetic wearers. Patients experience greater freedom of movement, reduced skin irritation, and improved aesthetic results. Custom coloring and texturing techniques ensure the prosthesis blends seamlessly with the patient's skin tone and texture, enhancing self-confidence and psychosocial well-being.

## Introduction

Midfacial defects, whether congenital or acquired, present a significant challenge for patient rehabilitation. Midfacial defects may involve the cheek, nose, upper lip, and the muscles beneath these areas. These defects can result from a range of factors, including injuries, burns, infections, surgical resection of tumors, radiotherapy, congenital abnormalities, or vascular malformations [1].

Basal cell carcinoma (BCC), formerly called basal cell epithelioma, is the most prevalent type of cancer in humans [2]. It is typically a slow-growing tumor, but it can be very damaging and cause significant disfigurement if not treated adequately or effectively [2]. Basal cell carcinoma occurring in the nasal vestibule is more prevalent among Caucasian individuals but rare in individuals of African and South Asian descent [3]. Originating from the basal cell layer of the epidermis, the disease is primarily caused by sun-induced skin damage, contributing to 90% of cases.

Unlike the one-third incidence of non-melanoma skin cancers among Caucasian individuals, Indian individuals experience these cancers in only 1-2% of cases [4]. This type of carcinoma often manifests in the upper central part of the face, particularly in sun-exposed areas such as the face and neck [3,5].

Treatment of basal cell carcinoma of the nasal area boasts a high cure rate exceeding 95%, though delays in seeking treatment can lead to cancer growth and potential disability [3,6]. Treatment approaches vary based on factors like cancer size, depth, and location, encompassing surgical removal, chemotherapy, and radiation [7]. Additional methods such as cryosurgery, Mohs micrographic surgery, electrodessication, and photodynamic therapy are also viable options for head and neck cancers [3,8].

After undergoing rhinectomy, patients may experience a major decline in their quality of life if appropriate surgical reconstruction or a prosthetic device is not quickly provided [9,10]. Prosthetic rehabilitation for nasal defects following trauma or surgery is well-documented [10]. The process of creating an extraoral prosthesis typically involves a series of stages: surgical, provisional, and definitive [11].

This clinical report aims to describe the fabrication of a custom-made definitive nasal prosthesis. It was crafted from polymethyl methacrylate resin, featuring a nose-piece secured using eyeglasses for retention.

## Case presentation

**Figure 1:**
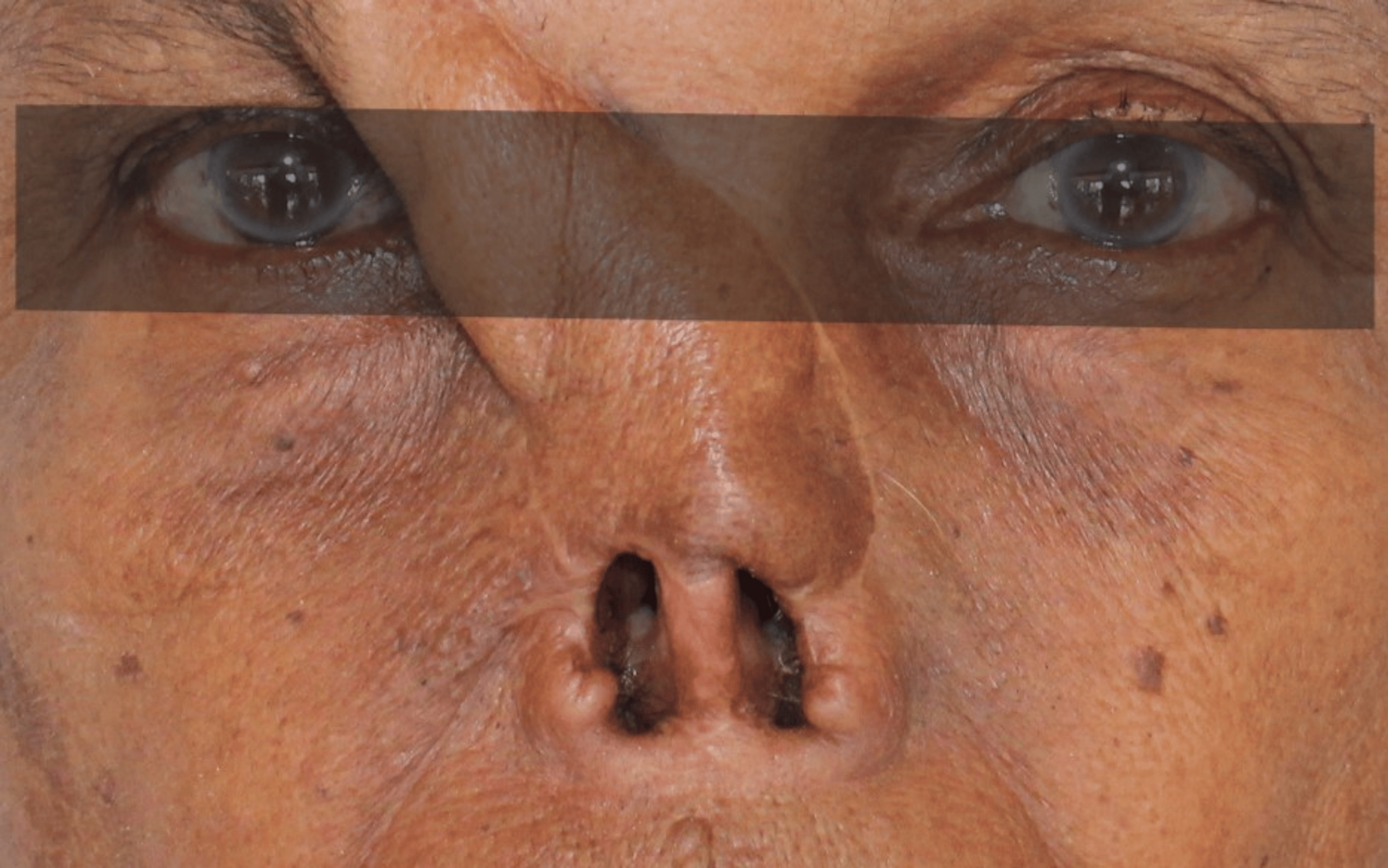
Nasal defect after partial rhinectomy due to basal cell carcinoma

A 64-year-old woman was referred to the Department of Prosthodontics for a nasal prosthesis following partial rhinectomy for basal cell carcinoma, seeking a nasal prosthesis to address facial disfigurement (Figure 1) and her lack of confidence to be among people. The surgery spared the bridge of her nose and nasal bones. She wore an interim prosthesis but expressed concern about facial disfigurement and discomfort and wanted a more definitive nasal prosthesis. Her medical history was irrelevant, apart from the partial rhinectomy done one year ago for basal cell carcinoma. Various prosthetic options, including acrylic resin and implant-retained silicone prostheses, were discussed. Due to financial constraints, she opted for an acrylic resin nasal prosthesis retained using eyewear.

**Figure 2:**
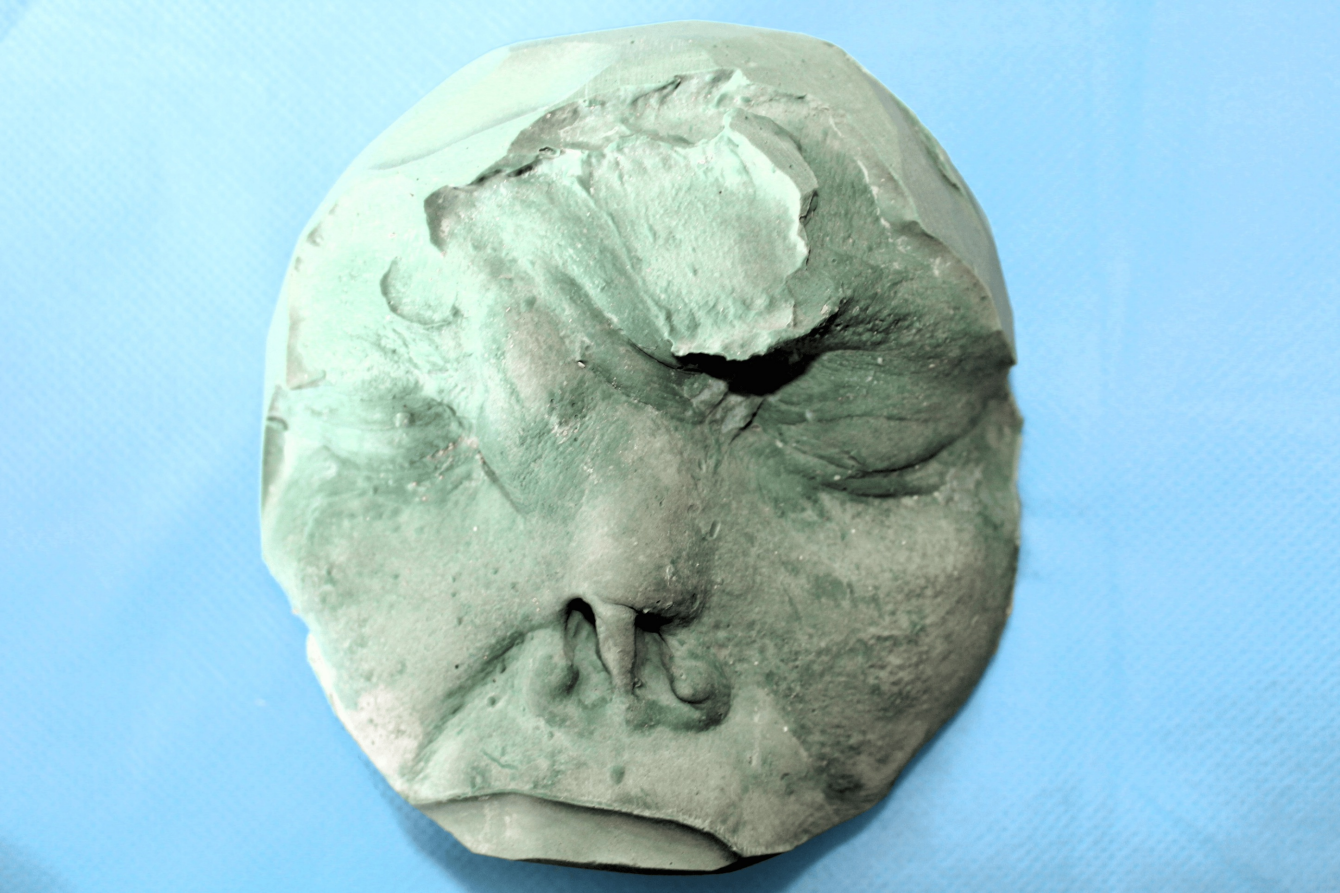
Working model of the defect

**Figure 3:**
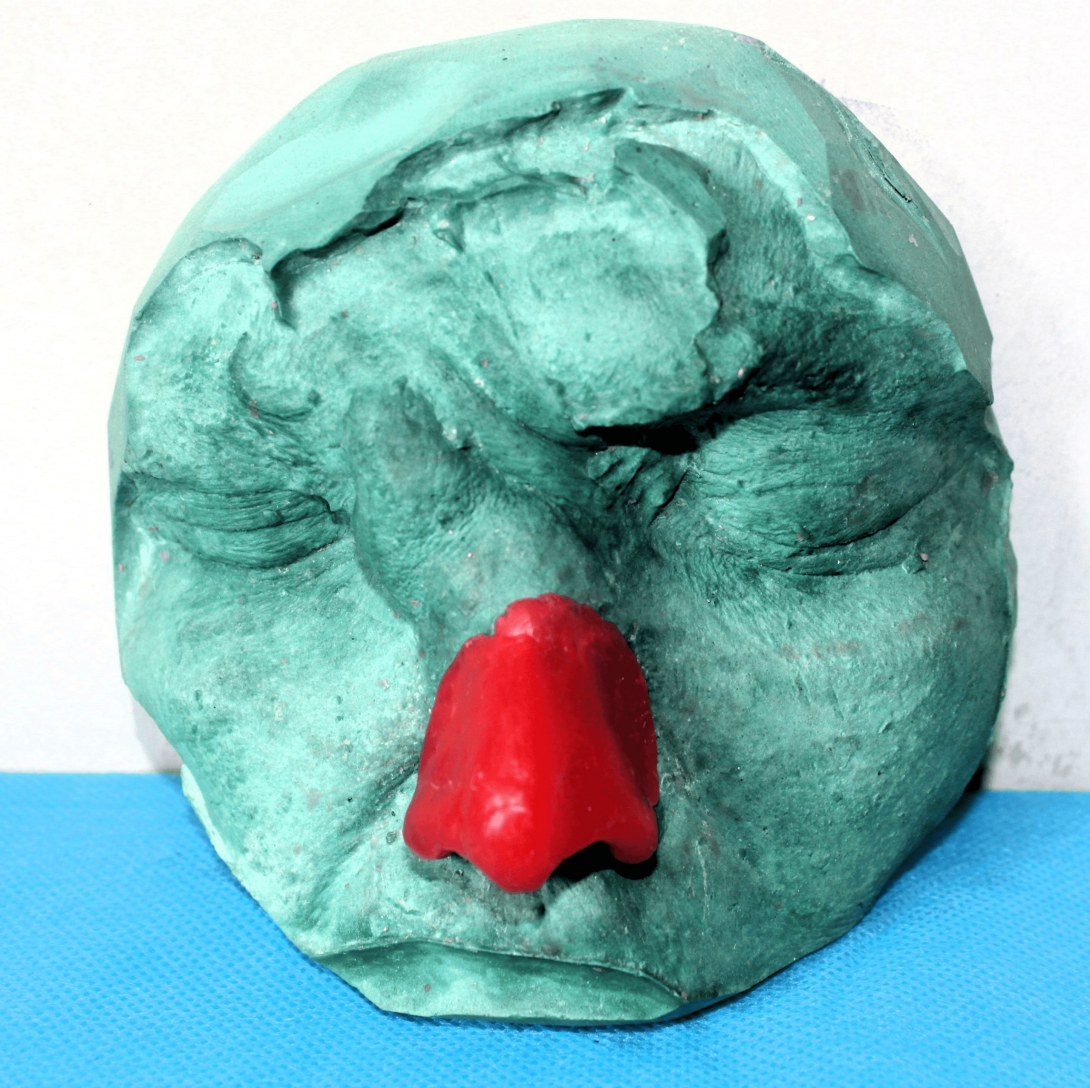
Wax pattern trial on the working model to assess the fit and adaptation of the borders

After thoroughly explaining the treatment outcome to the patient, a spectacle glass frame was chosen to support and secure the polymethyl methacrylate (PMMA) resin nasal prosthesis. The patient's eyebrows and eyelashes were covered with petroleum jelly. Moist gauze was applied to prevent material from entering the undercuts. An impression was made using a heavy body polyvinyl siloxane material (Zhermack Elite HD+, Zhermack SpA, Italy) in a semi-upright position. A cast (working model) was poured using type III dental stone (Goldstone) (Figure 2). A model of the prosthesis was shaped on the cast using No. 2 dental modeling wax. Esthetic contours were designed after taking into consideration the patient’s overall appearance and prior photographs (Figure 3).

The adaptation of the borders of the wax pattern on the patient’s face was carefully examined. The texture and contour of the tissues were evaluated using remaining anatomical landmarks and a preoperative photograph as a reference. Functional wax (Correcta wax, Kerr Corp., United States) was applied to the edges to ensure optimal adaptation with the underlying tissues. The wax model was then invested. After dewaxing, processing of the nasal prosthesis was done using clear PMMA resin material (DPI Heat Cure, Dental Products of India, Uttarakhand, India). Acrylic-based paints (Fevicryl, Pidilite Industries Ltd., India) were used to add intrinsic coloring to match the basic skin tone.

**Figure 4:**
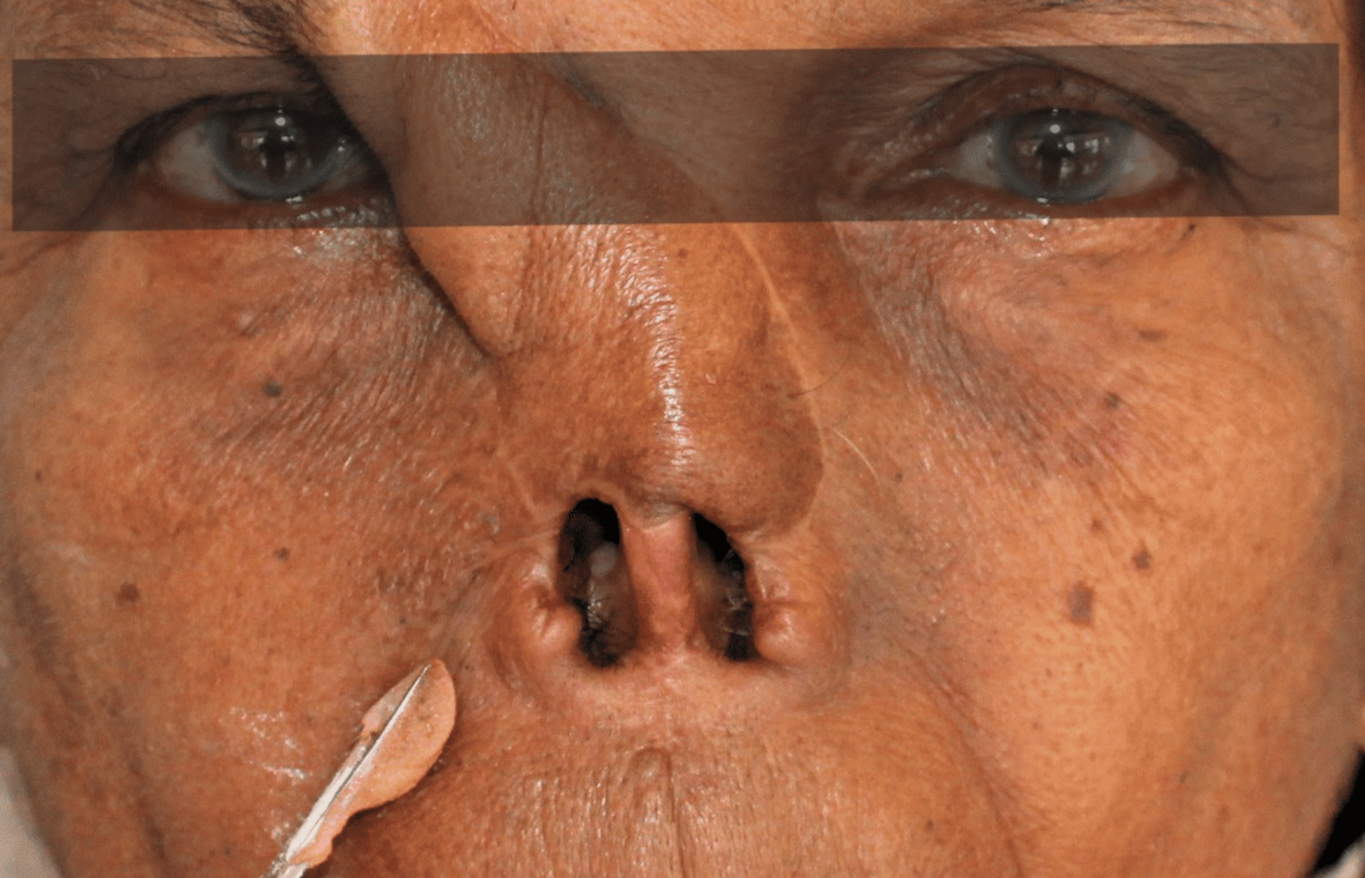
Extrinsic shade matching with acrylic-based paints to imitate the patient's skin tone

**Figure 5:**
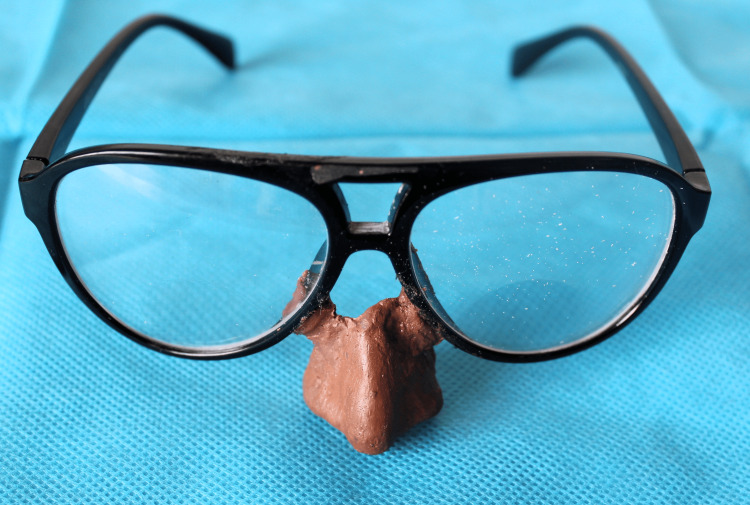
Finished acrylic nasal prosthesis retained with eyewear

A trial insertion of the nasal prosthesis was conducted. Extrinsic color mixing and matching was done to imitate the patient's skin tone using the same acrylic-based paints (Figure 4). Monopoly syrup was applied to the finished acrylic prosthesis with a camel hairbrush to make the extrinsic coloring water-resistant. The superior margins of the acrylic nose prosthesis were adapted meticulously to integrate with the eyeglass frame. The frame was used to enhance retention and cover the prosthesis margin. Finally, the frame was attached to the prosthesis (Figure 5). The placement of the prosthesis was demonstrated to the patient and then delivered. Detailed instructions regarding care and usage were provided to ensure proper maintenance.

**Figure 6:**
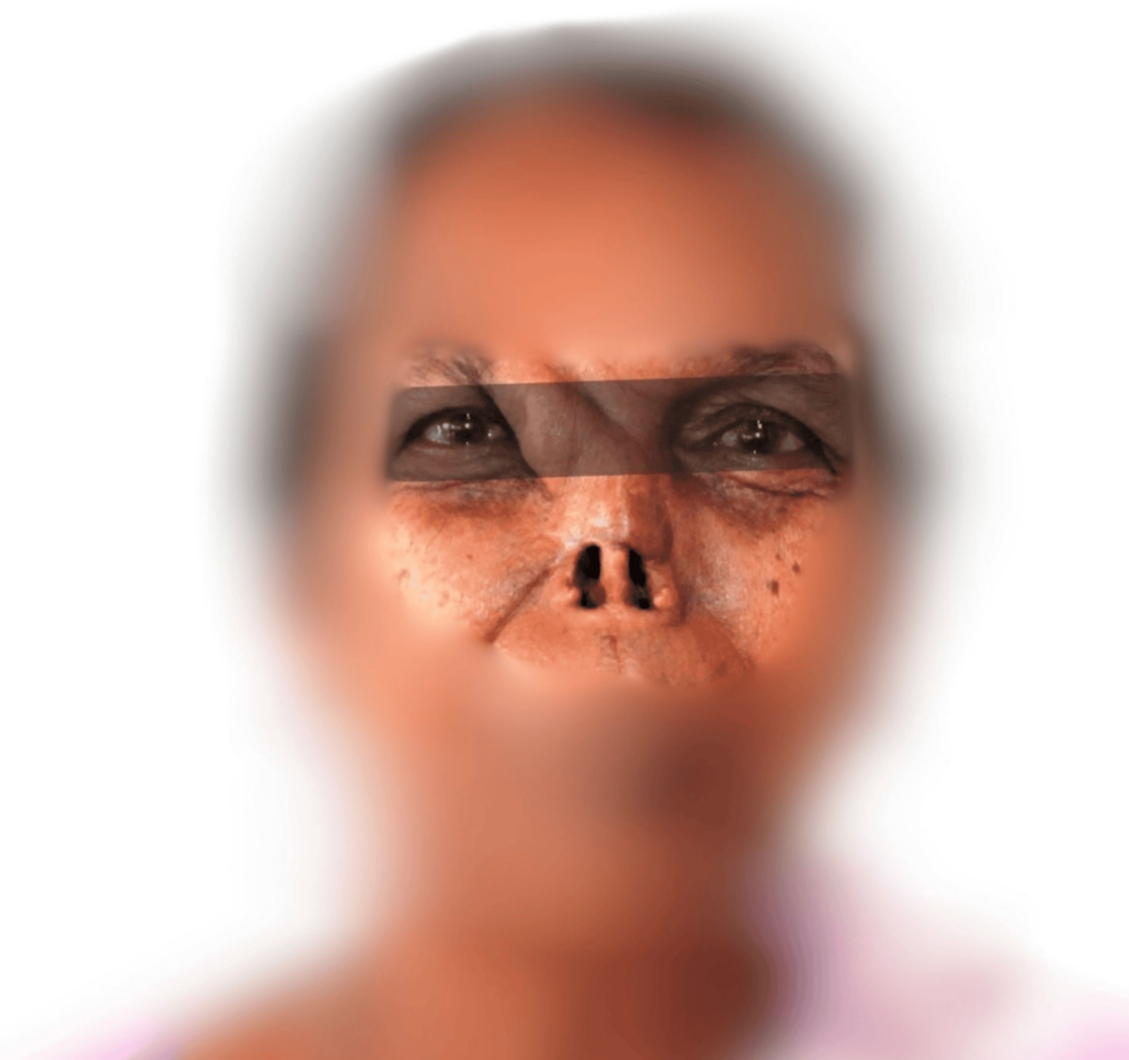
Frontal view of the defect before treatment

**Figure 7:**
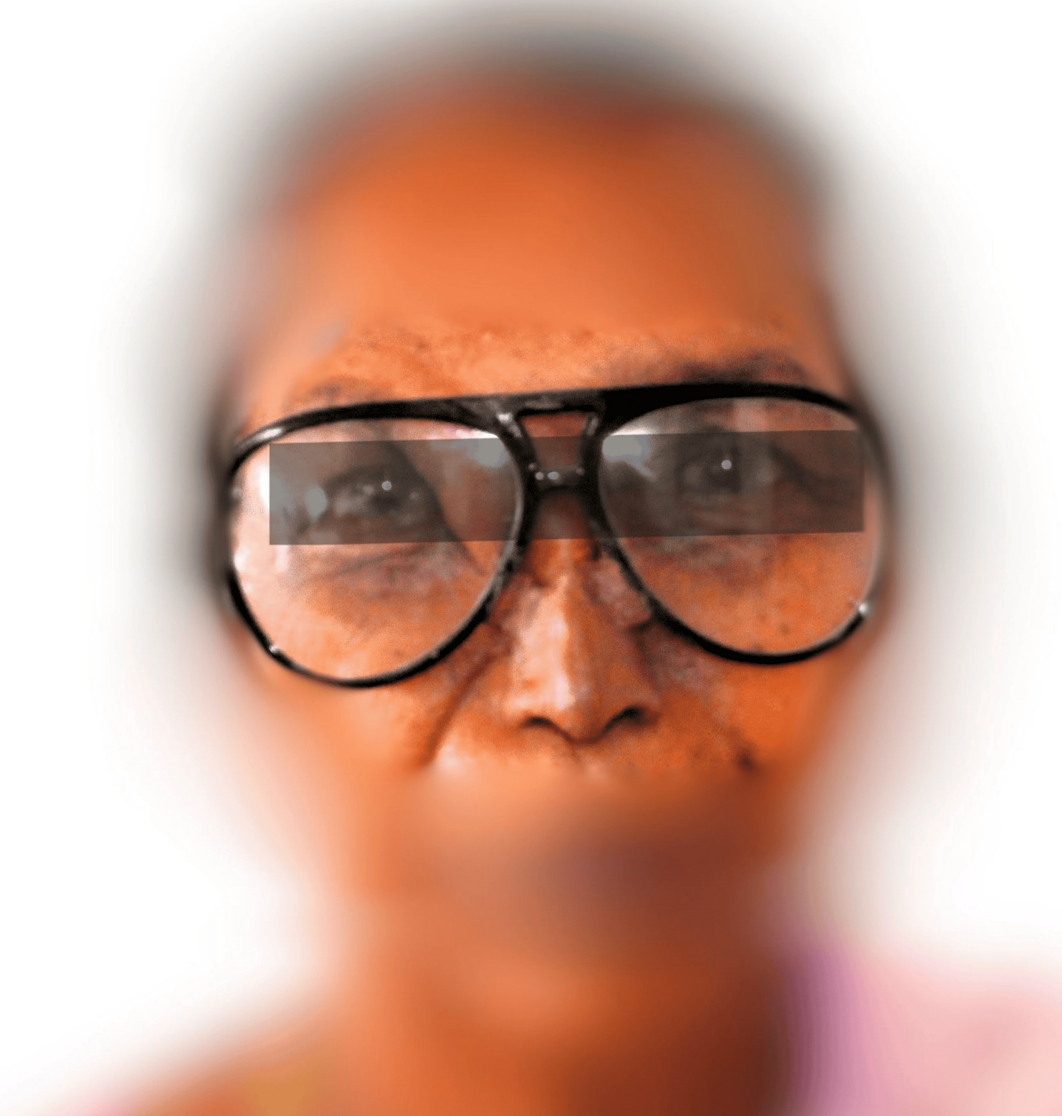
Frontal view after treatment with the eyewear retained nasal prosthesis

**Figure 8:**
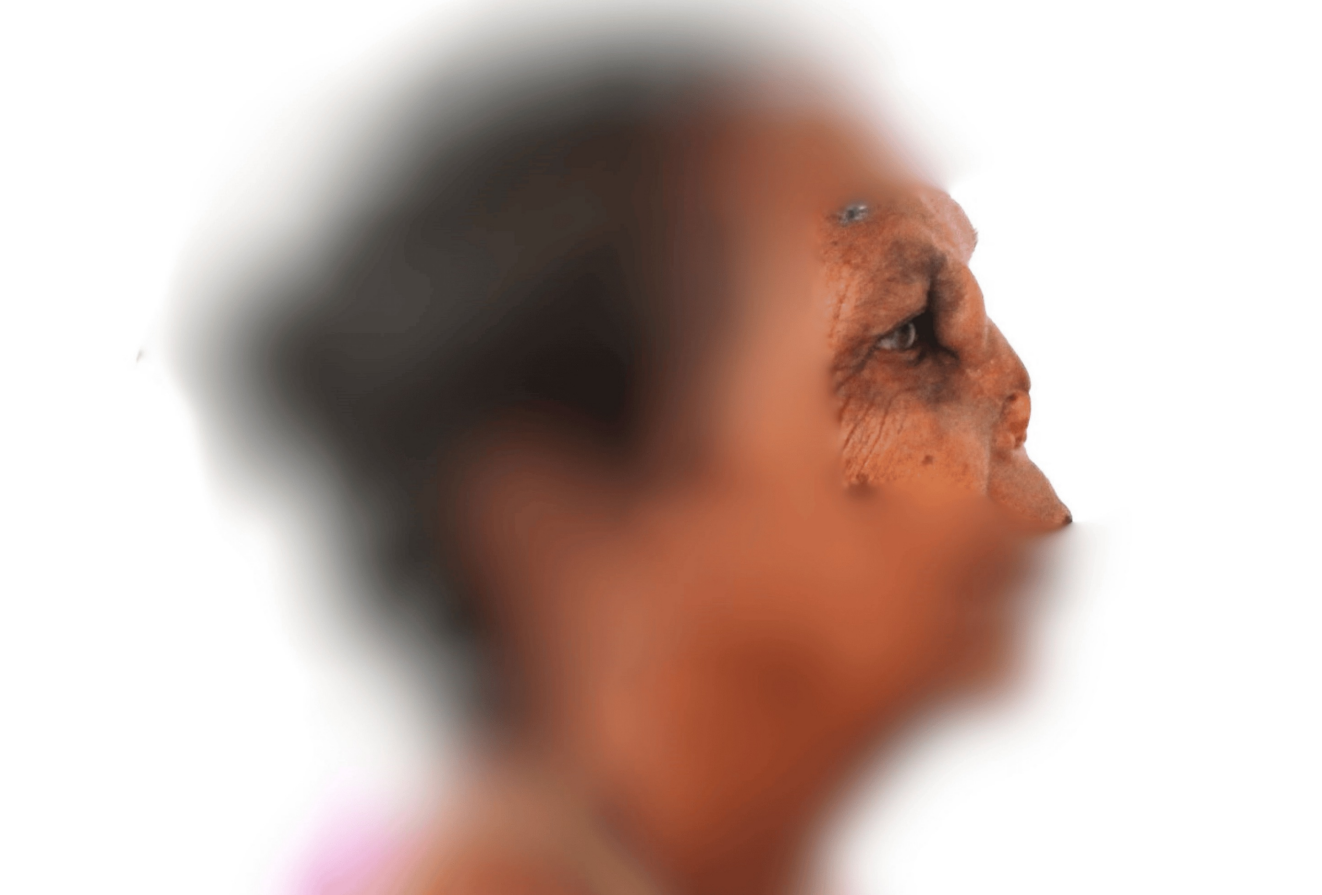
Pre-treatment lateral view of the defect

**Figure 9:**
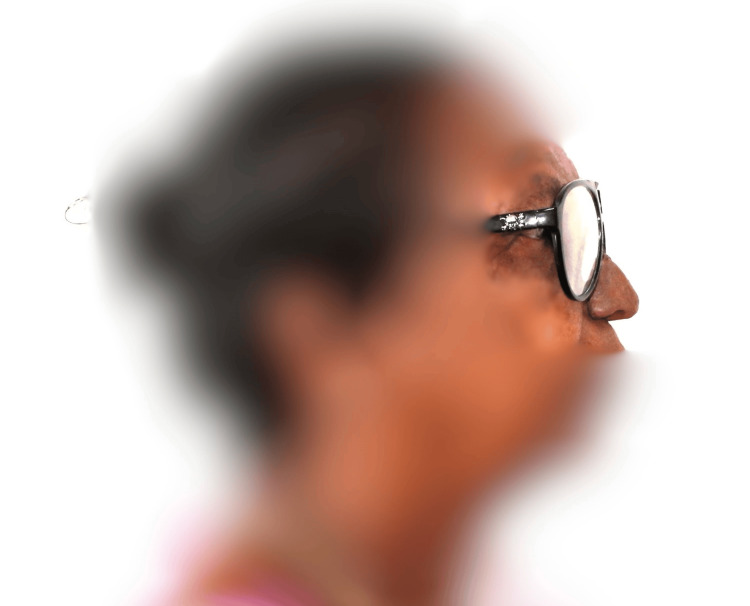
Post-treatment lateral view with the eyewear retained nasal prosthesis

The patient's first post-insertion adjustment was scheduled for the day after placement to ensure tissue health and alleviate any pressure points from the prosthesis. The prosthesis was found to be functioning normally during the follow-up evaluation conducted after four weeks (Figure 6, 7, 8, 9). The patient expressed satisfaction with the treatment outcome and reported a significant elevation in her mood as she felt confident attending social events with the prosthesis. The patient was advised to return for follow-up visits every three months to assess the fit of the prosthesis and to further evaluate the quality of life of the patient after wearing the prosthesis.

## Discussion

Facial defects, particularly nasal ones, significantly impact appearance due to the nose's prominent role in the facial profile. Surgical reconstruction or prosthetic rehabilitation, or a combination of both, is essential for restoring facial disfigurements, considering factors like defect location, size, and patient preferences [12]. When reconstructive surgery is extensive or risky, prosthetic rehabilitation becomes preferable due to potential recurrence, complexity, or aesthetic importance [13]. Patient education before surgery is crucial to manage expectations and postoperative care [14].

Historically, nasal prostheses relied on straps, intraoral extensions, or eyewear frames, which remain popular even today for their cost-effectiveness. Modern prosthetics mostly use adhesives for retention, offering ease of application and satisfactory hold, even though there is a mild risk of irritation and damage to the margins of the prosthesis during removal [15]. Other methods include utilizing anatomic undercuts, attachment to maxillary obturators [13], magnets [16], precision attachments, and endosseous implants [17].

Effective retention methods are crucial for satisfactory rehabilitation. In some cases, retaining nasal bones after surgery enhances eyeglass support and prevents dislodgement. Retention methods for facial prostheses vary; for example, mechanical retention using anatomic undercuts is advantageous when conditions are suitable but may be less effective in flat tissue beds [18]. In such cases, acrylic extensions can be made to the nasal floor for retention [19].

Materials like polymethyl methacrylate (PMMA) and silicone are commonly used for facial prostheses. PMMA, despite its rigidity and opacity, is suitable for nasal prostheses due to its low cost and ease of fabrication [20]. Techniques like painting monopoly syrup for color stability enhance the aesthetic appeal of PMMA prostheses [12].

Advancements in technology, such as implant-retained silicone prostheses and computer-aided design and computer-aided manufacturing (CAD/CAM), improve the precision and quality of nasal prostheses [21]. These methods allow for virtual planning and evaluation without traditional try-in appointments, even though they are not very cost-effective. Therefore, despite the limitations of resins in flexibility and aesthetics, PMMA remains a practical choice for nasal prostheses due to its affordability and ease of use.

## Conclusions

Prosthetic rehabilitation plays a crucial role in restoring function and appearance for patients with facial defects, particularly following surgeries for conditions like basal cell carcinoma. PMMA resin is favored in many settings due to its affordability and versatility in prosthetic applications. While it may have limitations in terms of flexibility and natural appearance compared to more advanced materials like silicone, PMMA remains a practical choice, especially in resource-limited settings where cost-effectiveness is paramount. Advances in materials and techniques continue to improve the precision and aesthetic outcomes of prosthetic rehabilitation, offering patients greater comfort and confidence in their daily lives.
